# Transition-metal-catalyzed C-S bond formation: recent developments and pharmaceutical applications

**DOI:** 10.3389/fchem.2026.1886794

**Published:** 2026-07-30

**Authors:** Saima Muneer, Nathalia da Silva Brito, Aqsa Kanwal, Ayesha Tariq, Nasir Rasool, João Modesto Brito, Rafaely B. C. Santos, Muhammad Imran, Sumbal Saba, Jamal Rafique

**Affiliations:** 1 Department of Chemistry, Government College University Faisalabad, Faisalabad, Pakistan; 2 Laboratory of Sustainable Synthesis and Organochalcogen (LabSO), Institute de Química, Universidade Federal de Goiás - UFG, Goiânia, Brazil; 3 Instituto de Química, Universidade Federal do Mato Grosso do Sul – UFMS, Campo Grande, Brazil; 4 Chemistry Department, Faculty of Science, King Khalid University, Abha, Saudi Arabia

**Keywords:** cooperative bimetallic, C–S bond formation, late-stage functionalization, transition-metals, ullmann-type C–S couplings

## Abstract

Due to widespread applications of sulfur-containing frameworks, the construction of the C–S bond has emerged as a useful tool for synthetic organic and medicinal chemists in recent years. Transition-metal-catalyzed carbon–sulfur (C–S) bond formation represents a cornerstone of advanced organic synthesis due to its pivotal role in pharmaceuticals, agrochemicals, and functional materials. This review provides a comprehensive and structured overview of recent developments in C–S bond formation, covering both classical late transition metals (Pd, Ni, and Cu) and a broad range of miscellaneous metals (Mo, Ir, Rh, Cr, Mn, Zn, and Sc). Palladium catalysis, typically supported by phosphine or N-heterocyclic carbene ligands, enables efficient thiolation of aryl halides using thiols, disulfides, or sulfur surrogates. Nickel catalysis as a cost-effective alternative to Pd, displaying complementary reactivity toward aryl, alkyl, and heteroaryl electrophiles, often under ligand-controlled or reductive conditions, while copper follows Ullmann-type C–S couplings and oxidative thiolations using thiols, sulfinates, or elemental sulfur. Beyond these, molybdenum catalysts promote dehydrative and borrowing-hydrogen thio-etherification of alcohols, whereas iridium-based photoredox catalysis converts sodium aryl sulfinates into thioesters and thioalkynes. This review highlights the high compatibility of these methodologies with drug-like molecules and the significance of these methodologies.

## Graphical Abstract

**Figure:**
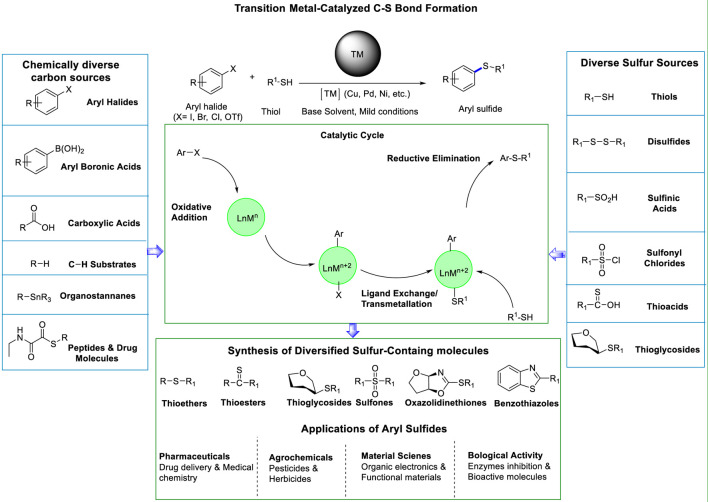

## 1 Introduction

**FIGURE 1:**
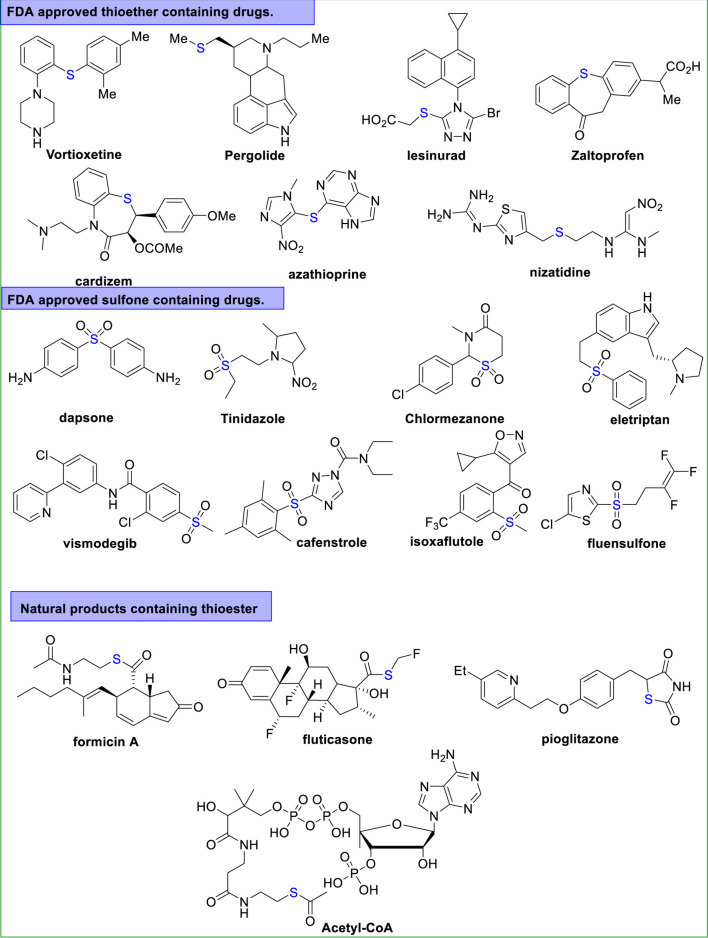
Selected FDA approved thioether, sulfones and thioesters containing drugs.

Pharmaceuticals, agrochemicals, and functional materials all contain sulfur-containing molecules, which are crucial to organic synthesis (Dunbar et al., 2017; Xiong et al., 2020). Organosulfur compounds constitute 20% of FDA-approved drugs, with over 200 drugs containing C−S bonds used to treat HIV, insomnia, gastroesophageal reflux, diabetes, cancer, schizophrenia, and Parkinson’s disease (McGrath et al., 2010; Scott and Njardarson, 2018). Because aromatic thioethers are found in a wide range of synthetic and natural organic compounds, the formation of C-S bonds continues to be one of the most significant issues in organic synthesis (Figure 1) (Uyeda et al., 2013). Thioethers, sulfones and thioesters play major roles in the biological processes, pharmaceuticals, and in the synthesis of organic products (Feng et al., 2016; Han and Küçükgüzel, 2022).

Conventional approaches as nucleophilic substitution, thiol–halide reactions, or acid-mediated thio-etherification, lead to harsh reaction conditions, limited substrate scope, poor chem-oselectivity (Schindler et al., 2025; Wei et al., 2022). Transition metals play a crucial role in carbon–sulfur (C–S) bond formation by enabling efficient cross-coupling reactions under controlled conditions (Wang et al., 2024). Most transition metal-catalyzed techniques now in use rely on costly and comparatively rare palladium-based catalysts, which necessitate standard ligand design for effective catalysis (Bhunia et al., 2017) Among them, palladium, nickel, copper, iron, and cobalt have been most widely studied, each showing distinct reactivity and selectivity profiles (Olaoye and Sejie, 2025). Pd uses Pd (0)/Pd(II) cycles with oxidative addition, trans-metalation, and reductive elimination, often via palladacycles for selective annulations/sulfenylation, offering high tolerance. Ni employs Ni(0)/Ni(II) or Ni(I)/Ni(III) for radical paths, enabling reactive, cost-effective couplings on hindered substrates. Cu relies on Cu(I)/Cu(III) or SET, coordinating sulfur sources for ligand-free, green syntheses with broad scope (Samanta et al., 2021).

**SCHEME 1:**
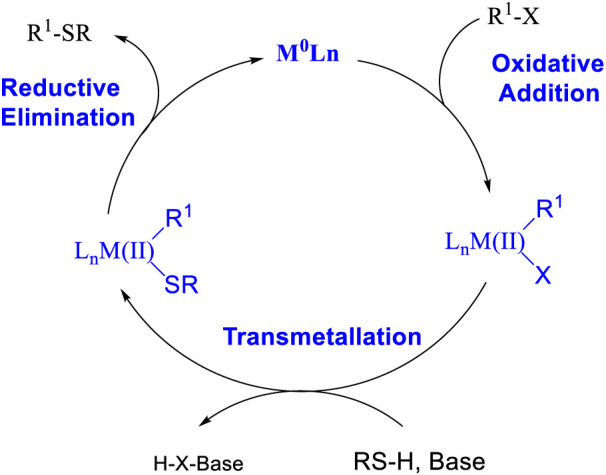
Mechanism for metal-catalyzed C–S bond-forming cross-coupling reaction.

The general mechanism of the metal-catalyzed C–S cross-coupling reaction has been shown in Scheme 1. Yet, carbon-sulfur bond formation remained challenging due to catalyst poisoning by sulfur sources (Lou et al., 2020). Recent breakthroughs in catalytic systems and cross-coupling strategies have addressed this issue, driving the development of sustainable methods for *S*-arylated organosulfur compounds (Debnath et al., 2025). With growing pharmaceutical, innovative approaches using diverse sulfur surrogates, novel ligands, and photoredox catalysis offer eco-friendly alternatives to traditional metal catalysis (Chen et al., 2022; Banik, 2024).

This review focuses on current developments in transition metal–catalyzed carbon–sulfur bond formation, with particular emphasis on the synthesis of thioethers, thioesters, sulfones, biologically relevant heterocycles, as benzothiazoles, benzo [*b*]thiophenes, thio-bridged frameworks, thioglycosides, and late-stage functionalization of bioactive scaffolds often through radical, photoredox, or cooperative bimetallic pathways. This work continues our ongoing research into exploring new sustainable methodologies for the formation for chalcogen compounds, and greener chemistry (Godoi et al., 2019; Haroon et al., 2021; Moraes et al., 2023; Rafique et al., 2016; Rocha et al., 2017; Saba et al., 2015; Saba et al., 2016; Sousa et al., 2024).

In this review, we systematically discuss catalytic strategies based on nickel, copper, and palladium, ligand selection, and reaction conditions that govern efficiency, selectivity, and substrate scope. Key developments include Ni- and Cu-catalyzed cross-couplings for aryl and alkyl thioether formation, metal-mediated thio-carbonylation and reductive coupling routes to thioesters, and photocatalytic sulfonylation protocols enabling access to sulfones (Tai et al., 2023). Also, other metals with a broad substrate scope were discussed (Huang et al., 2022). By comparing reactivity trends, mechanistic features, and practical applicability, this review provides a comprehensive perspective on how modern transition-metal catalysis has expanded the synthetic toolbox for efficient and sustainable C–S bond formation (Beletskaya and Ananikov, 2022).

### 1.1 Nickel catalyst

Nickel complexes have emerged as versatile and efficient catalysts for C–S bond formation owing to their strong metal–sulfur interactions and ability to operate under mild and sustainable conditions. N-heterocyclic carbene (NHC) ligands have shown high efficiency in aryl–thiol couplings, strongly influencing catalytic activity. Electron-rich, non-fluorinated NHC–Ni (II) complexes generally enhanced oxidative addition and facilitated of reductive elimination. Beyond traditional thermal catalysis, nickel has demonstrated remarkable adaptability in photoredox-assisted cycles and metal–ligand cooperative pathways, enabling C–S coupling reactions at room temperature, in air, and with low metal loadings. These strategies allow access to a wide range of sulfur-containing motifs, including aryl thioethers, alkyl thioethers, and thioesters, with broad functional-group tolerance.

**SCHEME 2:**
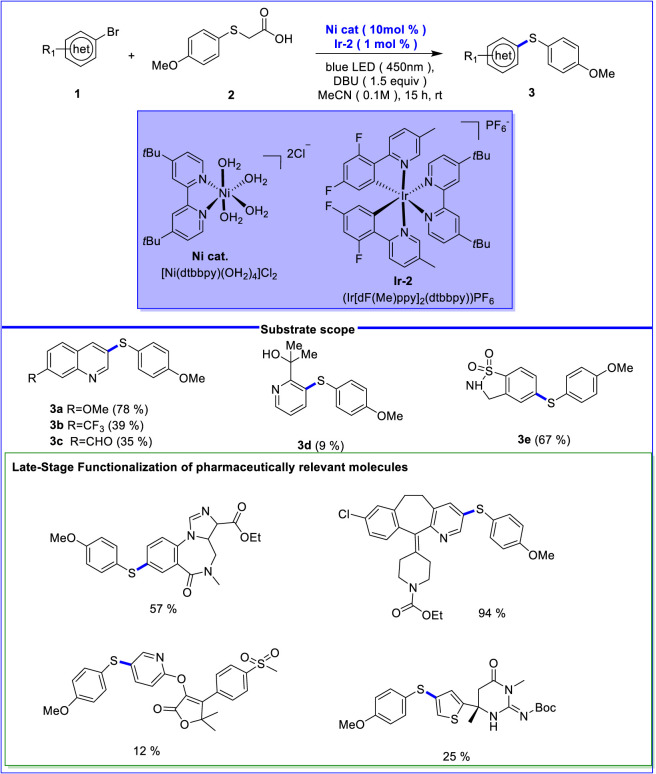
Nickel-catalyzed C–S cross-coupling for biaryl thioethers.

Christian *et al.* described the use of heteroaryl bromides (**1**) and 2-((4-methoxyphenyl)thio)acetic acid (**2**) in metallaphotoredox-catalyzed C-S cross-coupling to get biaryl thioethers (Christian, 2021). This technique makes it possible to couple building blocks containing reactive groups, such as nitrogen heterocycles, which are important in the pharmaceutical industry. Sulfur’s variability in oxidation states enables the adjustment of molecular properties, making it vital for drug development, as exemplified by amoxicillin and esomeprazole, which demonstrate its pharmaceutical relevance. The reaction yielded moderate to good results, with specific yields noted 78% for -OMe substituted quinoline and 39% for -CF_3_. Other substrates, including aldehydes and chlorinated quinolines, also demonstrated varying yields under the optimized conditions, indicating the method’s adaptability. Under the reaction conditions, both pyrimidine and alcohol were tolerated, however the alcohol yield was only 9%. Notably, 2-bromoheteroaryls achieved yields of 59% and 54%, respectively (Scheme 2).

**SCHEME 3:**
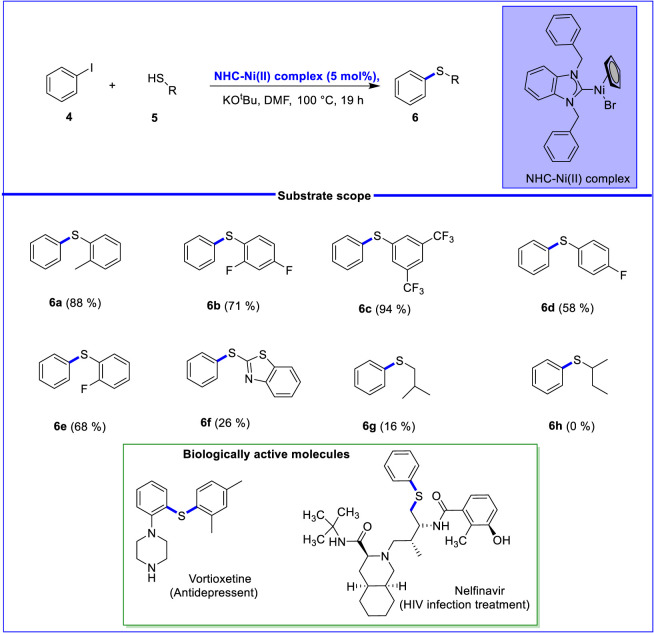
Synthesis of aryl sulfides.

Over the past 20 years, *N*-heterocyclic carbene (NHC) ligands have been thoroughly investigated in organometallics and catalysis to create highly catalytically active species in processes like cross-coupling, metathesis, dehydrogenation, hydrogenation, and other industrially significant reactions (Mata et al., 2007; Peris, 2017; Valdés et al., 2018). Romano *et al.* investigated C–S couplings using Ni (II) complexes with *N*–heterocyclic carbene ligands, achieving high conversions (>99%) with thiophenol derivatives (**5**), while aliphatic substrates yielded low conversions (Jaimes et al., 2023). The catalytic performance varied based on substituent groups; fluorine at para or meta positions resulted in 68%–58% conversions, while the meta–derivative showed no conversion. Notably, a meta-positioned–CF_3_ group afforded a conversion of 94%, whereas the para-position led to only 39% conversion (Scheme 3).

**SCHEME 4:**
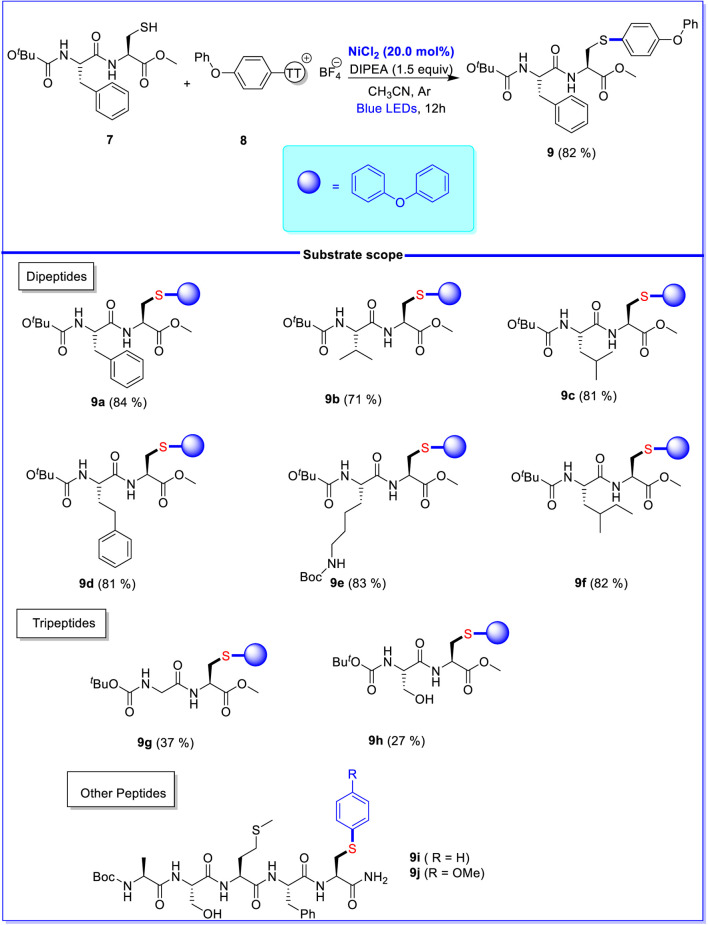
Synthesis of arylated cys (cysteine) containing dipeptides.

Qin *et al.* developed the visible light-induced nickel-catalyzed selective *S*-arylation of peptides (**7**) by exogenous photosensitizer-free photocatalysis (Qin et al., 2023). The arylated product was synthesized with an 84% yield using 20 mol% NiCl_2_ and 1.5 equivalents of DIPEA in acetonitrile under blue light for 12 h. Substituting NiCl_2_ with NiBr_2_ or Ni (OTf)_2_ yielded lower results, while variations in NiCl_2_ concentration affected the yield negatively. Since para-substituted phenyl rings with electron-neutral, electron-rich, and electron-poor groups all successfully produced *S*-arylated peptides in moderate to good yields (36 %–79%), the electronic nature of substituents had little effect on product production. Furthermore, more complicated substrates with several functional groups were likewise compatible and produced the required products in yields ranging from 30% to 78%. Acetonitrile proved superior to other solvents like methanol and DMF. The presence of DIPEA was critical, as alternative bases resulted in poor yields (Scheme 4).

Thioesters are very important intermediates in life processes, pharmaceuticals and organic materials (Franke and Hertweck, 2016). Wen-Peng Mai *et al.* reported the nickel-catalyzed thioesters from aromatic and hetero aromatic amides and disulfide (Mai et al., 2024). This combines with inexpensive metal and easily available substrates to produce a number of thioesters in moderate to good yields by the breaking of *C*-*N* and *S-S* bonds. On the aromatic ring, electron-donating substituted groups like methyl and tert-butyl produced a product yield of 70% and 74%, respectively. Additionally, electron-withdrawing groups including CF_3_, CN, NO_2_, and COOMe were also compatible, and the related compounds were effectively produced in moderate yields. Compared to 1,2-di(thiophen-2-yl)disulfane, 1,2-bis(furan-2-ylmethyl)disulfane performed poorly, and the corresponding product was only produced in a 42% yield.

**SCHEME 5:**
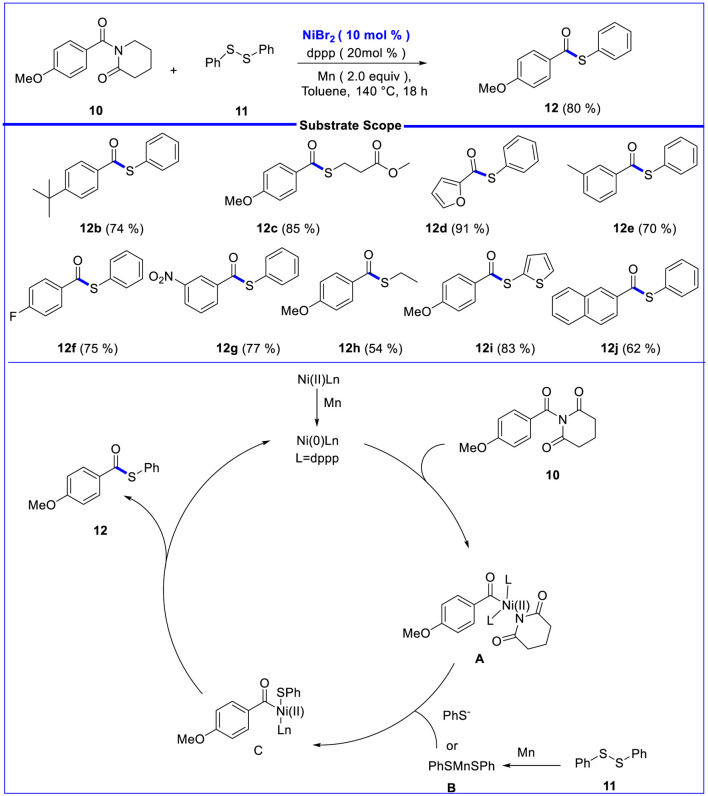
Synthesis of thioesters from aromatic amides and disulfides.

The mechanism describes a major feasible method for the coupling of amides with disulfides via *C-N* cleavage, which was catalyzed by nickel. First, substrate *C-N* bond is inserted by the catalyst Ni (0) (Ln), which results in species **A**. Manganese aids in the cleavage of the disulfide to produce the aryl thiolate ion or Mn-chalcogenolate intermediate **B** based on related studies. After that, complex **B** transmetalates with intermediate **A** to produce intermediate **C**. Ni (0) species was then renewed for the subsequent catalytic cycle, and the desired product was obtained through reductive elimination (Scheme 5).

**SCHEME 6:**
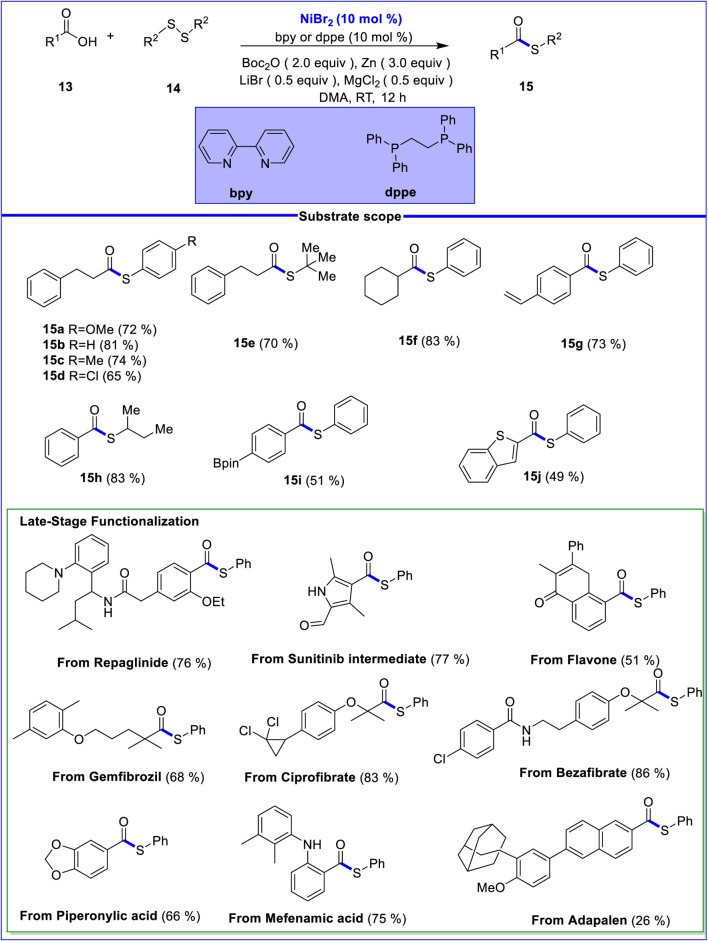
Synthesis of thioester by nickel catalyst.

Wu *et al.* revealed a reductive cross coupling reaction between carboxylic acids (**13**) and a variety of diaryl disulfides, such as electron-rich, electron-poor, sterically hindered, and heteroaryl substrates (**14**), that is catalyzed by nickel to access thioesters (**15**) (Wu et al., 2023). To effectively produce structurally varied thioesters, the reaction takes place at room temperature with broad functional group tolerance. Functional groups including esters, vinyl, and formyl were well tolerated in the protocol, while benzoic acids with electron-neutral, electron-withdrawing, and electron-donating groups shown good compatibility. Nevertheless, at the reaction conditions, substrates with free–OH and–NH_2_ groups were incompatible. Additionally, the reaction was tolerant of a wide range of substrates, including sterically hindered disulfides. In the end, this effective technique may be used for late-stage functionalization to create various thioesters, providing a practical and easy way to create carbon-sulfur linkages (Scheme 6).

**SCHEME 7:**
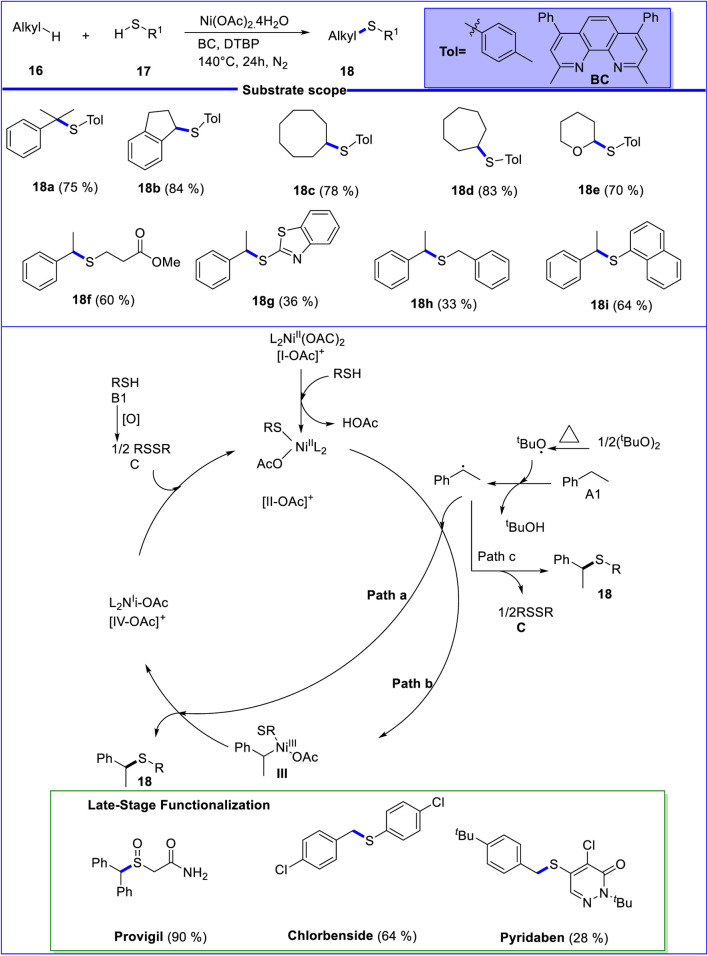
Synthesis of alkyl thioethers by Ni catalyzed oxidative dehydrogenative coupling.

Ni catalyzed oxidative dehydrogenative coupling provides an efficient, directing group free method for sp^3^ C-S bond formation (Ni et al., 2012). Liu *et al.* revealed the oxidative dehydrogenative coupling of alkane **(16)** with thiol (**17**) for the production of C (sp^3^)-S bonds, which is accelerated by nickel (Liu et al., 2021). Sterically hindered bathocuproin (BC) is used as ligands to stabilize the nickel-center and avoid catalyst poisoning. Ni-catalytic cycles are pathways a and b. Ni-complex [II-OAc]+ is created when [I-OAc]^+^ and thiol exchange ligands, this produces complex [IV-OAc]^+^ after reacting with the alkyl radical [I-OAc]^+^ (path **a**) is then produced by the SET process of [IV-OAc]+ with disulfide **C**. Additionally, route **b** cannot be eliminated since the alkyl radical contributes to [II-OAc]+. This is followed by the C–S reductive elimination of [III-OAc]^+^ to release the product (**18**) and [IV-OAc]+, which then combines with disulfide **C** via the SET process to create [I-OAc]^+^. A metal-free SET procedure is used in path **c.** Alkyl radical and tert-butyl alcohol are produced when a hydrogen atom in **A1** is captured by a tert-butyl oxygen radical. Alkyl radicals react with **C** to produce alkyl thioether (**18**) (Scheme 7).

**SCHEME 8:**
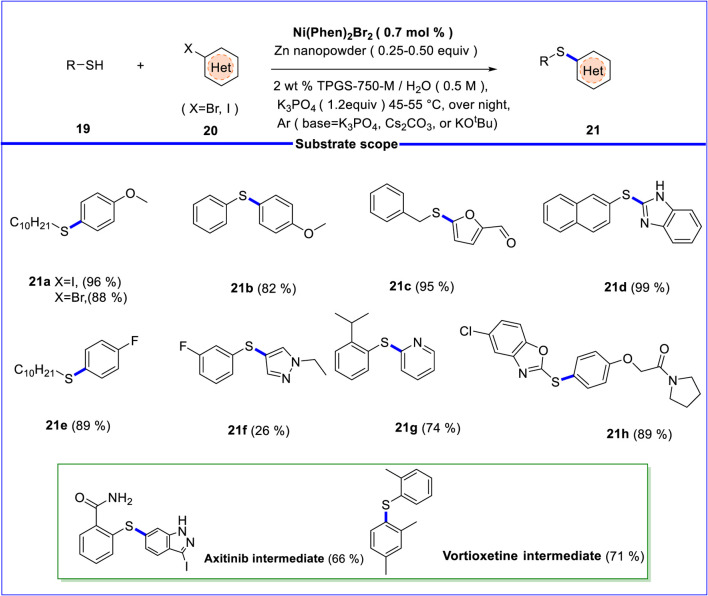
Synthesis of thioethers through Ni (Phen)_2_Br_2_.

Yu *et al.* reported a mild and scalable nickel-catalyzed C-S coupling using micellar catalysis in recyclable water (Yu et al., 2021). It efficiently converts diverse heteroaryl halides and thiols into thioethers in good yields. It replaces toxic organic solvents with recyclable water and uses low catalyst loadings. The protocol works efficiently with diverse thiols, including challenging alkyl thiols (**19**) and aryl iodides/bromides (**20**), yielding thioethers with yields that are good to outstanding (Scheme 8).

NHC ligands are strong, tunable *σ*-donors, but rarely used in C-S coupling. Phthalimide-based NHC-Ni^II^ complexes with 2-Ni (*n*-Bu substituent) proved the most active, giving up to 96% yield. Conversions were higher for alkyl substrates with more steric hindrance than for those with less impediment. Within 30 min, thiophenols with electron-withdrawing groups underwent over 90% conversion, demonstrating their strong reactivity. The catalyst’s weak activity against heterocyclic thiols, on the other hand, suggests that its substrate scope is limited. Its superior activity reflects the higher electron-donating ability of the *n*-Bu NHC ligands.

**SCHEME 9:**
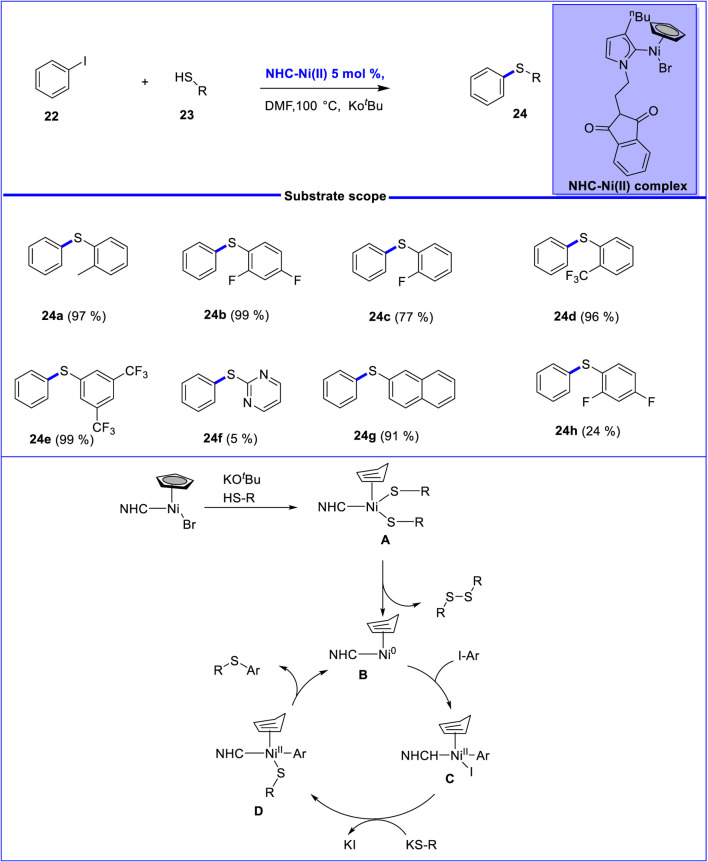
C-S couplings catalyzed by [(NHC)Ni(Cp) (Br)] complexes.

Rodríguez-Cruz *et al.* reported the C-S cross-coupling catalyzed by a series of easily accessible, NHC-Ni^II^ complexes bearing phthalimide and Cp ligands facilitates C-S coupling of iodobenzene (**22**) with various thiols (**23**) (Rodríguez-Cruz et al., 2020). Such C-S bond formation is valuable for synthesizing biologically important molecules (Boyd, 2016; Evano et al., 2013). A potential mechanism initiates with thiolate coordination and Cp de-aromatization form an intermediate that eliminate disulfide to give Ni (0) which react with iodobenzene (**22**) and undergoes ligands exchange and reductive elimination to produce the thioether (**24**) and regenerate the catalyst (Scheme 9).

**SCHEME 10:**
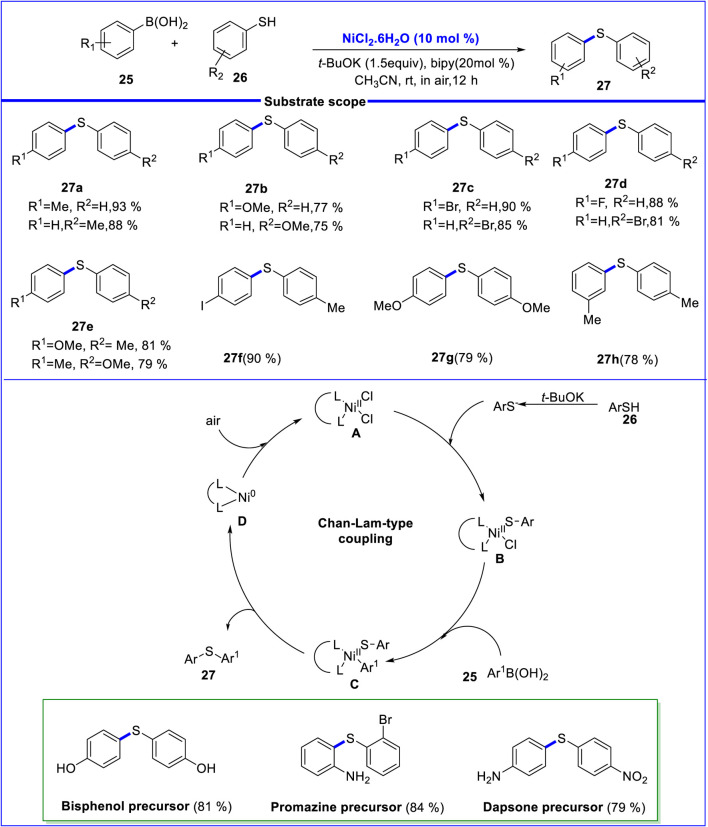
Synthesis of diaryl sulfides.

Cancer, Alzheimer’s, Parkinson’s, IDS, neoplastic, HCV, diabetic, and parasite disorders are among the many medical conditions where diaryl sulfides are utilized. According to Bhowmik *et al.* mono aryl substituted diaryl sulfides were produced in high to exceptional yields by nickel-catalyzed cross-coupling of aryl boronic acids (**25**) with thiophenols (**26**) using NiCl_2_.6H_2_O and 2,2′-bipyridine (Bhowmik and Yadav, 2020). **A**was obtained by coordinating the bipy ligand with NiCl_2_. The species **B** is created when a chloride from **A** is displaced by the deprotonated thiol. Arylboronic acid trans-metalation yields species **C**, which is then eliminated reductively to produce the product (**27**). The catalytic cycle is continued by oxidizing the Ni (0) complex **D** to Ni (II) in air (Scheme 10).

**SCHEME 11:**
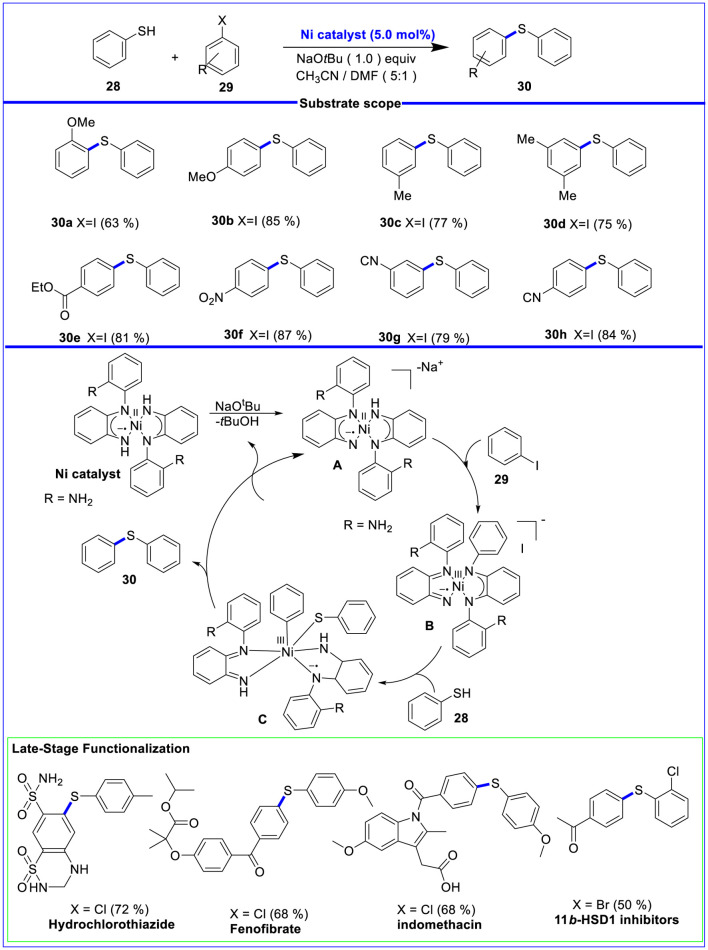
Synthesis of diaryl sulfide by Ni-catalyst.

Sikari *et al.* described the use of metal-ligand cooperativity in the presence of NaO*t*Bu to catalyze C-S cross-coupling under mild conditions (Sikari et al., 2019). The mechanism of action is that the reaction occurs by *t*-BuO -induced deprotonation of the catalyst, producing **A,** which in turn reacts with Ph -I to create an intermediate **B**. The increase in the electron density facilitates easy activation of Ar that is I which oxidizes Ni (II) to Ni (III) with the simultaneous transfer of an electron through the deprotonated ligand. Subsequent coordination and proton transfer to the oxidized ligand give the intermediate **C**, which reacts with reductive elimination to form a diaryl sulfide product (Scheme 11).

**SCHEME 12:**
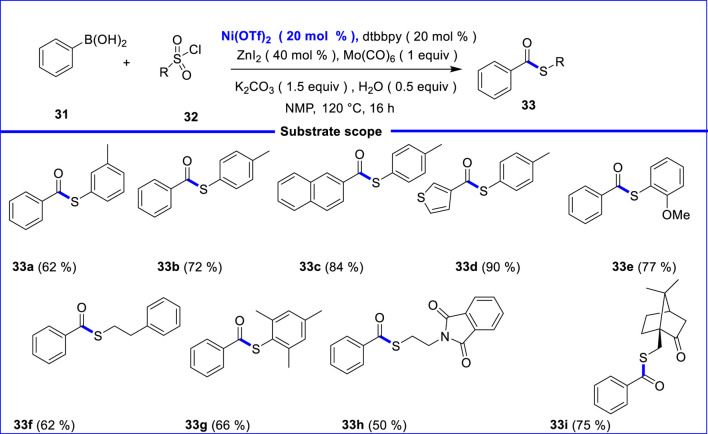
Synthesis of thioesters by Ni(OTf)_2_ with aryl boronic acid and sulfonyl chloride.

Thioesters are crucial biochemical intermediates that have garnered a lot of interest due to their quick biological characteristics (Ilardi et al., 2014). Thioesters are helpful building blocks in organic chemistry that are often used as air-stable, easily handled acyl donors in a variety of processes to access different ketones^47^, aldehydes (Miyazaki et al., 2004), esters (Os’ kin et al., 2009) and amides (Ueda et al., 1981). Qi *et al.* described the synthesis of thioesters using the nickel-catalyzed thio-carbonylation of aryl-boronic acids (**31**) with sulfonyl chlorides (**32**) (Qi et al., 2020). With the optimal reaction conditions, the substrate scope of aryl boronic acids for this thio-carbonylative transformation electron-donating or electron-weakly withdrawing aryl boronic acids were reacted with thioesters in good to excellent yield, whereas strongly electron-withdrawing groups (CN, CF_3_) led to proto deboronation (Scheme 12).

**SCHEME 13:**
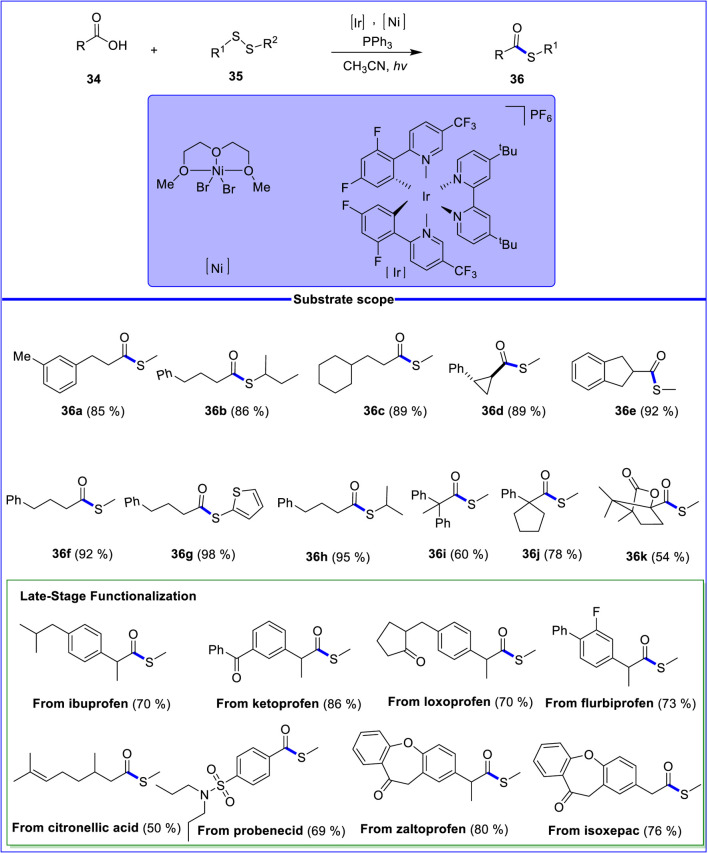
Synthesis of thioester via nickel-catalyzed sensitized electron transfer.

Huamin *et al.* reported the radical thio-esterification by sensitized electron transfer catalyzed by nickel (Wang et al., 2023). DMDS reacted with primary and secondary carboxylic acids (**34**) to yield methyl thioesters (**36**) in excellent yields (50%–92%). Electron-rich aromatics, keto groups, and cyclic alkyls were well-tolerated (56%–92%). Tertiary acids were also easily converted in moderate yield (54%–78%). The protocol was also general and yielded moderate to good results (50%–75%) with aryl acids that had electron-giving or -taking groups, including ortho-substituted counterparts (Scheme 13).

**SCHEME 14:**
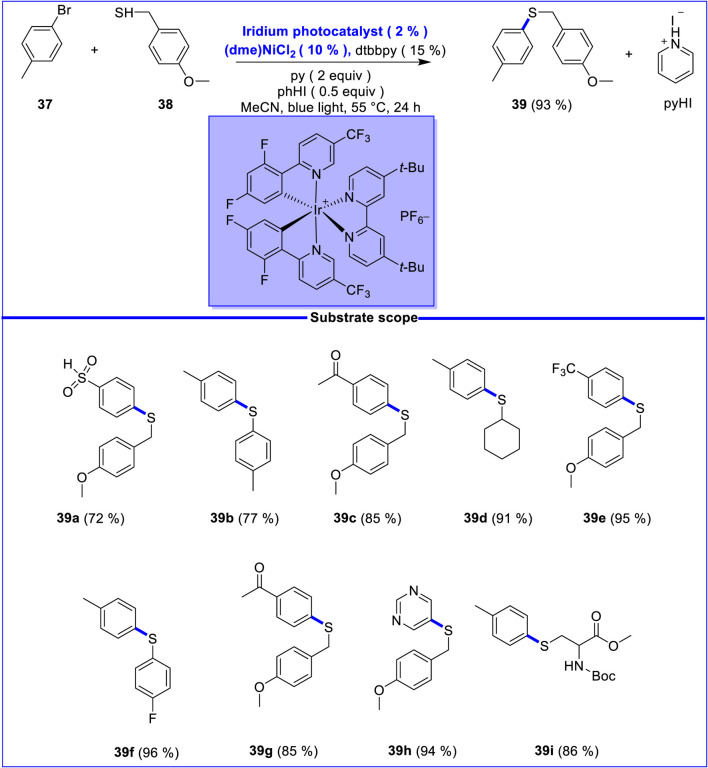
Synthesis of thioethers.

Thioethers are often found in medicines and natural bioproducts (Jarrett, 2015). Qin *et al.* described the C-S cross-coupling catalyzed by iridium photocatalyst (Qin et al., 2021). It has been successful to use a photoredox catalyst in combination with transition metal catalysis (Zhang et al., 2015a), particularly to induce nickel-catalyzed carbon-heteroatom bond synthesis under moderate circumstances utilizing straightforward and affordable ligands (Lim et al., 2018). C-S cross-coupling between 4-bromotoluene (**37**) and thiol (**38**) was made possible by the addition of pyHI (Pyridinium hydroiodide) and a slightly higher temperature of 55 °C. The best approach worked effectively with aryl bromides that had electron-donating and electron-withdrawing groups, primarily aryl heterocycles. The approach also worked well with aryl and alkyl thiols. Additionally, the amino acid cysteine has a high yield, suggesting possible use in the synthesis of biomolecules (Scheme 14).

### 1.2 Copper catalyst

Copper complexes represent a highly versatile, economical, and mechanistically diverse class of catalysts for carbon–sulfur bond formation, offering efficient alternatives to precious-metal systems. Across a wide range of transformations including diaryl thioether synthesis, electrophilic thiolation, sulfonylation, *S*-arylation, radical cyclization, *S*–*S* bond cleavage, and thioglycosylation—copper catalysts consistently deliver moderate to excellent yields under relatively mild conditions (Zhu et al., 2015).

Notably, Cu-catalyzed systems demonstrate broad substrate scope, tolerating aryl iodides and bromides, organostannanes, sulfinates, thiols, xanthates, disulfides, carbohydrates, and complex drug-like molecules, thereby enabling late-stage functionalization and gram-scale synthesis. The applicability of copper catalysis extends to photoredox and radical-mediated mechanisms, where Cu (I) complexes act as efficient and sustainable alternatives to Ru- or Ir-based photocatalysts due to their long-lived excited states, strong reducing ability, and low toxicity. copper–ligand cooperation allows access to radical, ionic, and photoredox pathways, making ligand choice a powerful handle to control reactivity and functional-group tolerance. These features enable visible-light-driven sulfonylation and site-selective *S*-arylation of sensitive substrates such as thiosugars and biologically relevant scaffolds (Wang et al., 2015; Zhu et al., 2024).

### 1.3 Role of ligands in copper-catalyzed C-S formation

Liu *et al.* revealed the coupling of sodium sulfinates (**41**) and aryl iodides (**40**) catalyzed by Cu(OAc)_2_, which is DABCO-promoted diaryl thioether production (Liu et al., 2020). Additionally, a potential catalytic mechanism involving DABCO (1,4-Diazabicyclo [2.2.2]octane) was described, indicating that the crucial step in this coupling process is the synergistic reduction of sulfinate by Cu (II) and DABCO. Some electron-withdrawing groups did not clearly affect the reactivity to produce the corresponding yields of 77% and 93% from the reactions of 4-Cl and 4-CF_3_ benzene, electron-donating groups at the para position reduced the yields to 52%–67%, with the exception of 4-CN substituted iodobenzene, which was obtained with a yield of 49%.

**SCHEME 15:**
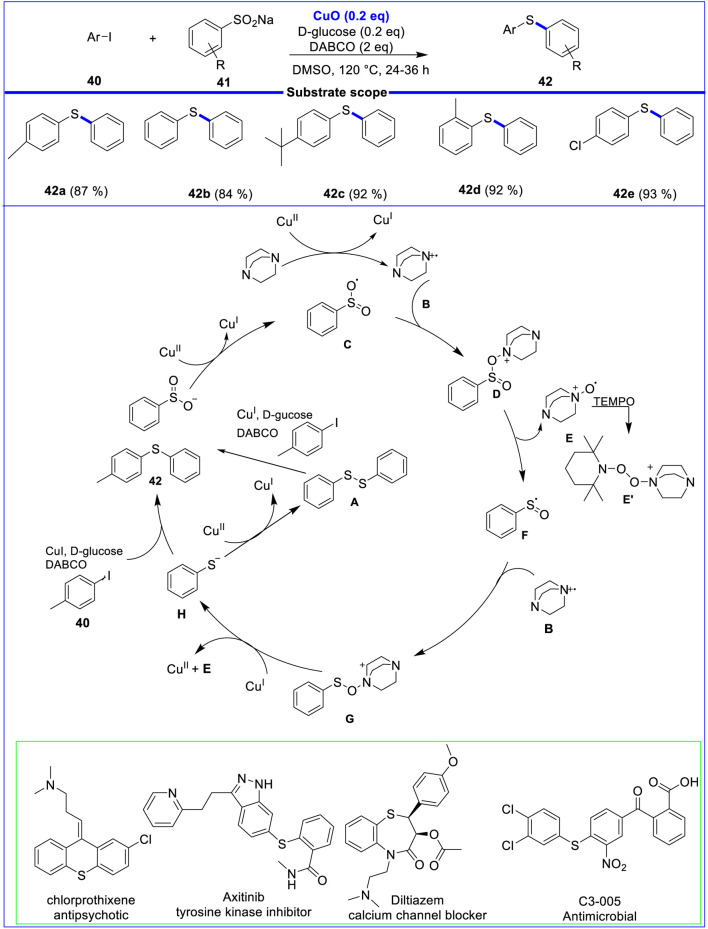
Synthesis of diaryl thioether.

Initially, Copper (II) oxidation generate DABCO radical cation **B**. Then, sodium benzenesulfinate oxidized by copper (II) to produce benzenesulfinate radical **C**, which then combine with **B** to generate DABCO-sulfinate. DABCO-sulfanolate **G** was produced when the reduced sulfinyl radical **E** combine with **B**, again following the leaving of DABCO *N*-oxide radical **D**. Copper (I) was oxidized to produce thiolate **H**, which then undergoes a coupling reaction with four-iodotoluene (**40**) to provide the final diaryl thioether product (**42**). Disulfide can spontaneously occur as an intermediate in the presence of thiolate, and **A** can constantly combine with four-iodotoluene (**40**) to produce thioether (**42**) (Scheme 15).

**SCHEME 16:**
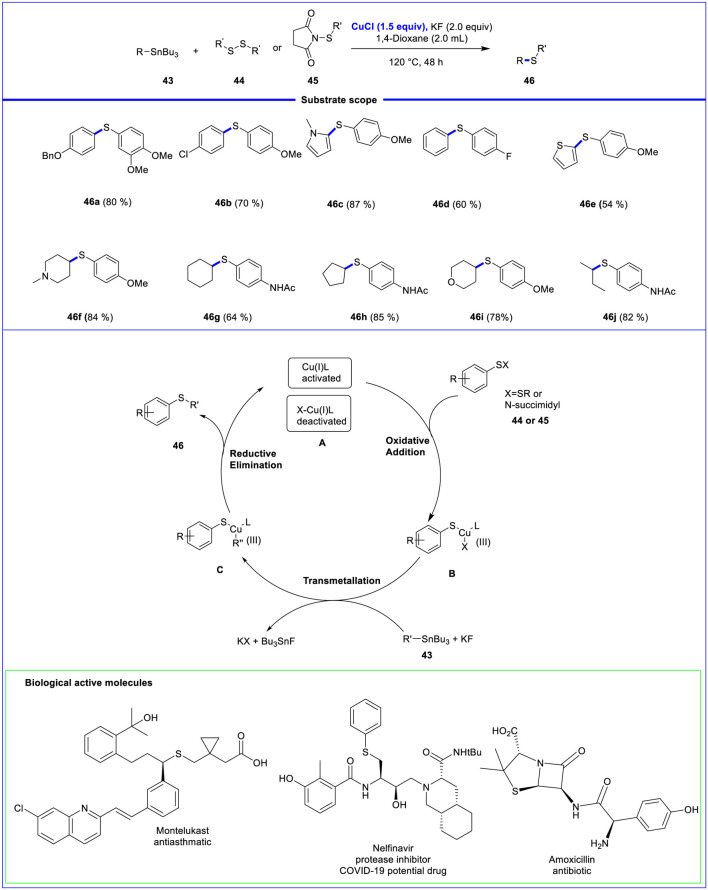
Ligand-free Cu(I) mediated electrophilic thiolation of organostannanes with sulfur electrophiles.

Zhu *et al.* described an electrophilic thiolation of organostannanes (**43**) with sulfur electrophiles (**44**) mediated by Cu (I) without the need for a ligand (Zhu et al., 2020). Sterically congested substrates and some alkyl organostannanes produced relatively lower yields, electron-rich and less sterically hindered aryl organostannanes often produced the best yields. Several typical alkyl stannanes effectively participate in the Cu (I) -mediated electrophilic thiolation as an example of substrate scope. For instance, at mild circumstances, tributyltin methyl, tributyltin ethyl, and tributyl (benzyl)stannane easily couple with sulfur electrophiles to produce the corresponding alkyl aryl sulfides in good to outstanding yields. Furthermore, sterically hindered secondary alkyl stannanes have moderate reactivity, demonstrating the methodology’s wide applicabilityThe oxidative addition product LCu (SR)X B is first produced when the Cu (I) species combines with electrophilic sulfur reagents RS−X (X = SR, *N*-succimidyl). Organostannanes undergo base-assisted transmetalation with product LCu(SR)X **B** to generate Cu (III) intermediate **C**, which subsequently goes through reductive elimination to provide the final product (**46**) along with regeneration of the deactivated X−Cu (I) L, such as RS−Cu species (Scheme 16).

**SCHEME 17:**
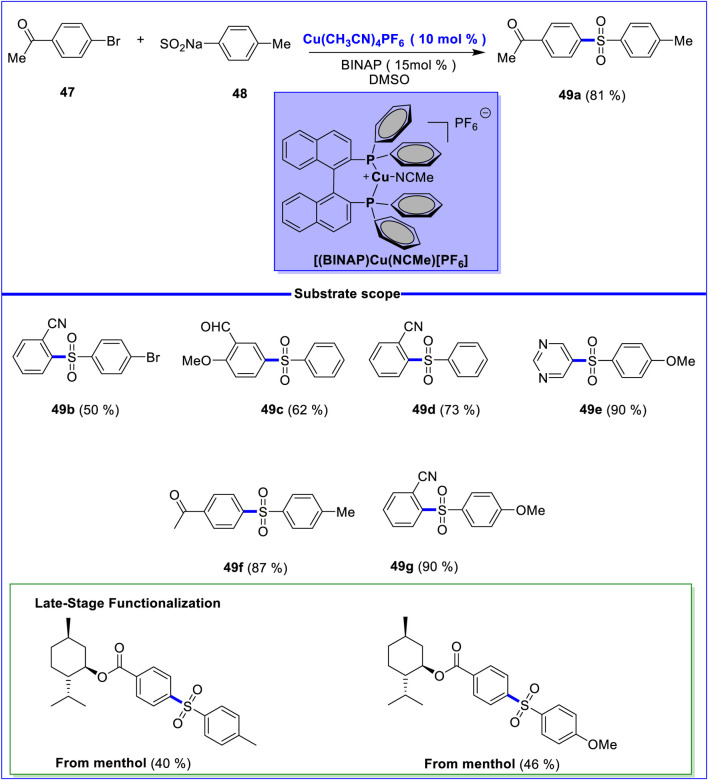
Sulfonylation of aryl halides by visible light/copper catalysis.

Organ-sulfones are versatile chemical building blocks widely utilized in synthetic and medicinal chemistry (McReynolds et al., 2004; Noshi et al., 2007). Yan *et al.* described the use of visible light and copper catalysis to sulfonyl-ate aryl halides (**47**) (Yan et al., 2021). Most photo catalysts have challenges of toxicity, being expensive, scarce of metals and not being stable in photo catalysis, which limits their use. The copper complexes have proved to be good substitutes in transformations of the visible light as they possess long excited state lifetimes, high reducing power, and relaxed ligand behavior. Cu (I) complexes, especially, are an effective way to promote electron transfer and a cost-efficient replacement of iridium or ruthenium catalysts. The product was obtained in 81% using 4-bromoacetophenone (**47**) in reaction with sodium 4-methylbenzenesulfinate (**48**) in Gram scale. The procedure also allowed alteration of biologically important molecules such as the *L*-menthol analogs (Scheme 17).

**SCHEME 18:**
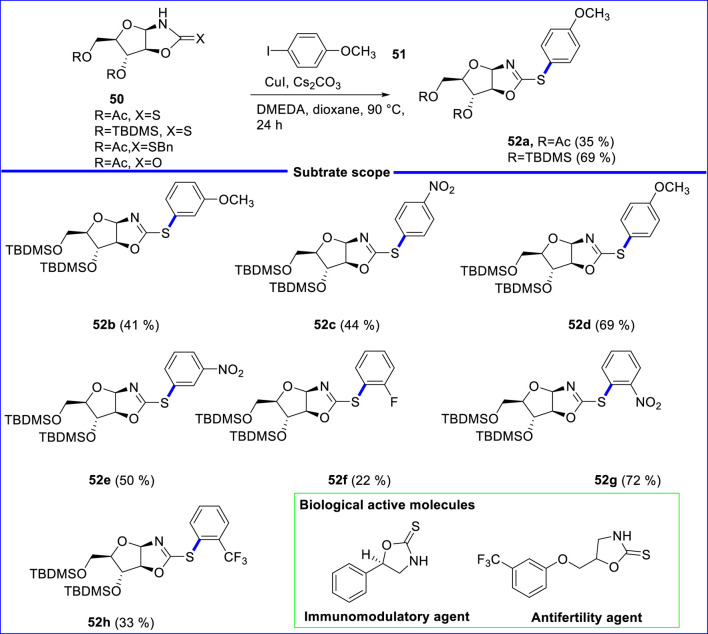
Copper-catalyzed *S*-arylation of furanose-fused oxazolidine-2-thiones.

Interesting biological characteristics of carbohydrates annulated with heterocycles include the selective inhibition of glycoside hydrolase *O*-GlcNAcase (OGA) or the simultaneous inhibition of OGA and cholinesterases, as well as the inhibition of the fructose transporter protein GLUT5 (Velueta-Viveros et al., 2022). Kederien˙*et al.* described CuI/DMEDA-catalyzed *S*-arylation of carbohydrate-anchored 1,3-oxazolidine-2-thiones (**50**) with aryl iodides (**51**) at 60 °C–90 °C afforded chiral oxazolines (**52**) in moderate to fair yields. (Kederienė et al., 2022). Applicability to various carbohydrate-anchored OZTs was demonstrated by extending the process to *d*-xylose and *d*-ribose oxazolidine-2-thiones with ortho-substituted iodobenzenes, yielding a range 45%–58% indicating the influnce of steric hindrance on the coupling efficiency. Overall the protocol demonstrated good compatibility with structurally diverse carbohydrate scaffolds while maintaining moderate reactivity towards sterically demanding substrates (Scheme 18).

**SCHEME 19:**
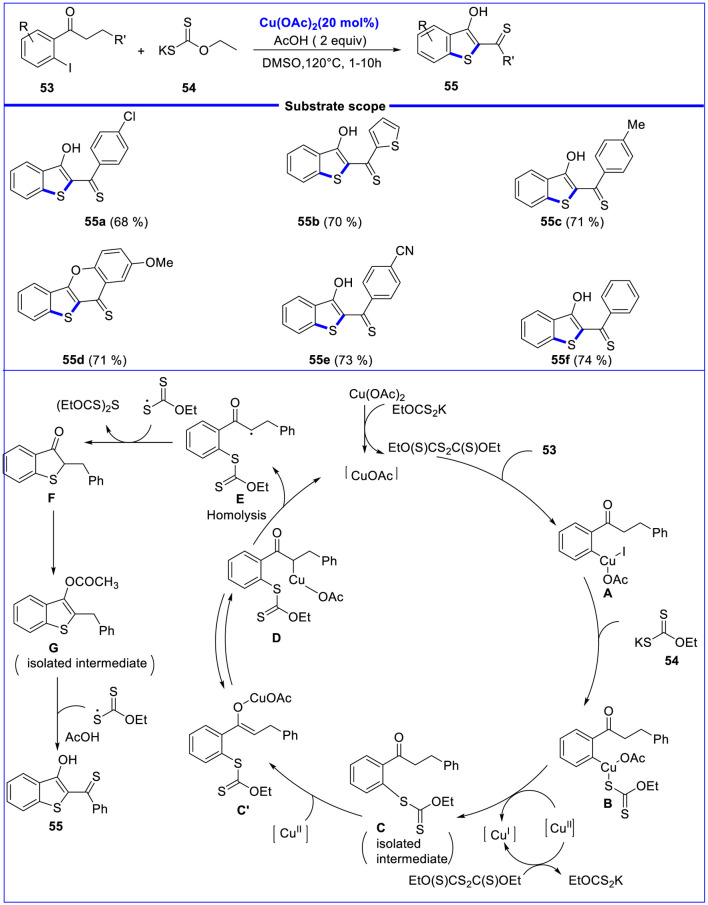
Copper-catalyzed domino synthesis of multi-substituted benzo [b]thiophene.

Numerous scientific domains, such as materials chemistry and the medical sciences, use multi-substituted 3-hydroxy benzo [b]thiophene (Guglielmi et al., 2019). According to Sundaravelu *et al.* xanthate was used as a sulfur surrogate in the copper-catalyzed domino synthesis of multi-substituted benzo [b]thiophene via radical cyclization (Sundaravelu et al., 2021). Cu promoted the radical cyclization of two-iodophenyl ketones (**53**) with xanthate (**54**), resulting in somewhat high quantities of tetracyclic lubinalbin analogues and multi-substituted benzo [b]thiophenes (**55**). Cu (I) oxidative addition, xanthate ligand exchange, and reductive elimination are all part of the process. The xanthate dimer may oxidize this Cu (I) to Cu (II), which will then react with the xanthate ester’s keto group to generate metal–enol complex **C**’. To produce **D**, the intermediate may go through keto-enol tautomerism. The radical intermediate **E** may eventually result from a homolytic cleavage of intermediate **D**. As a result, in situ-generated xanthate radicals cleave the xanthate ester to form the thieyl radical, which then cyclizes to give **F**. Through keto-enol tautomerism, the intermediate **F** should produce the stable aromatic intermediate **G**. Under the reaction conditions, the methylene group in intermediate **G** may change into a thiocarbonyl group, resulting in the product 3-hydroxybenzo [b] thiophenethione (**55**) (Scheme 19).

**SCHEME 20:**
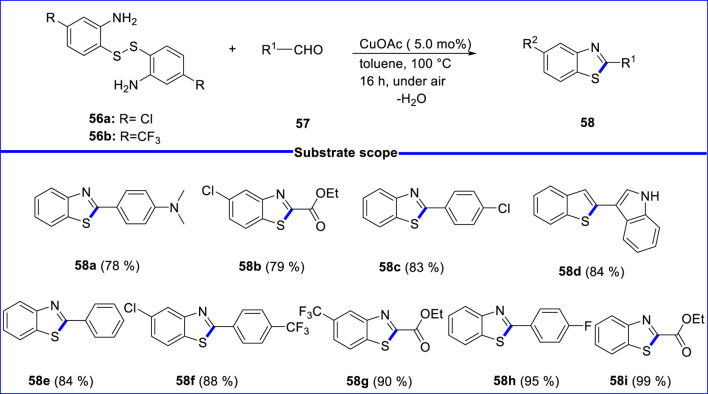
Synthesis of benzothiazoles through Cu-catalyzed *S-S* bond cleavage.

Because of their many biological functions and fluorescent characteristics, benzothiazoles are adaptable scaffold structures (Irfan et al., 2020; Tang et al., 2020a). Sulfur-sulfur (S-S) bond cleavage in organic processes often necessitates a stoichiometric excess of a reducing reagent (Jones et al., 2020) Although the catalytic cleavage of S-S bonds has been achieved (Groendyke et al., 2019), catalytic breakage and subsequently efficient catalytic carbon-sulfur (C-S) bond synthesis still present interesting tasks (Cheng et al., 2021; Taniguchi, 2022) Keisuke minami *et al.* described the synthesis of heterocyclic spiro compounds and benzothiazoles using Cu-catalyzed S-S bond cleavage and C-S bond formation (Minami et al., 2022). The reaction between 2,2′-dithiobis (benzenamine) (**56**) and formaldehyde (**57**) took place under the optimum conditions of CuOAc as the catalyst, 100 °C, in the presence of air to yield 1,3- benzothiazole (**58**) at 77% yield. Notably, at the optimized reaction conditions, a range of aromatic aldehydes were well tolerated, resulting in moderate to good yields of the respective benzothiazoles. In yields of 84%, 69%, and 85%, respectively, benzaldehyde, *p*-methoxy-benzaldehyde, and *p*-methyl-benzaldehyde produced the required compounds (Scheme 20).

**SCHEME 21:**
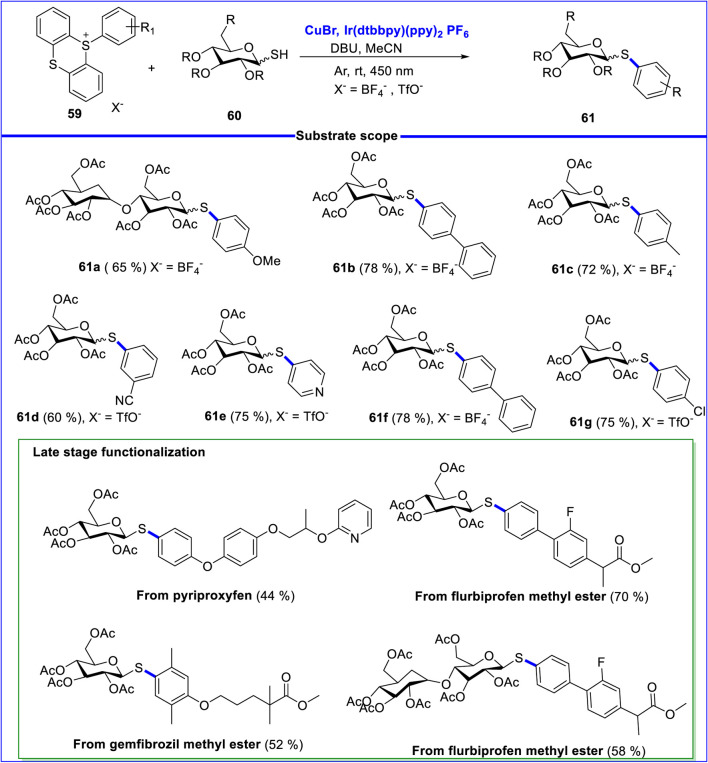
Copper (I)-mediated site-selective *S*-arylation of 1-thiosugars with aryl thianthrenium salts.

Aryl *S*-glycoside derivatives, as the mimetics of *O*-glycosides, are widely applied in the construction of biologically active oligosaccharides and glycoconjugates (Das and Mukhopadhyay, 2016; Xiao et al., 2016). Feng *et al.* reported a copper(I)-mediated photoredox protocol for the selective *S*-arylation of 1-thiosugars using aryl thianthrenium salts. The methodology proceeded under mild conditions and provided the desired aryl thioglycosides in moderate to excellent yields. (Fang et al., 2024). A photoreduction-oxidation Cu (I) method allows the production of aryl thioglycosides of aryl-thianthrenium salts (**59**) and 1-thiosugars (**60**) (with 32%–78% yields in the mild conditions of blue-light). The procedure is tolerant to a variety of substituted halogens, and can perform late-stage functionalization of natural products and drugs. It can work with disaccharide and mono-saccharides and with complex molecules, and it can give moderate to high yields of products, and gram-scale reactions demonstrate the practical usefulness of the strategy in the context of drug discovery (Scheme 21).

### 1.4 Palladium catalyst

Pd-catalyzed methodologies remain indispensable for the synthesis of sophisticated sulfur-containing molecules in medicinal and heterocyclic chemistry. Palladium acts as a highly adaptable catalyst in organic syntheses involving C-S bond formations and annulations primarily through mechanisms featuring oxidative addition, palladacycle intermediates, and reductive elimination, with differences in its oxidation states and the use of co-catalysts.

**SCHEME 22:**
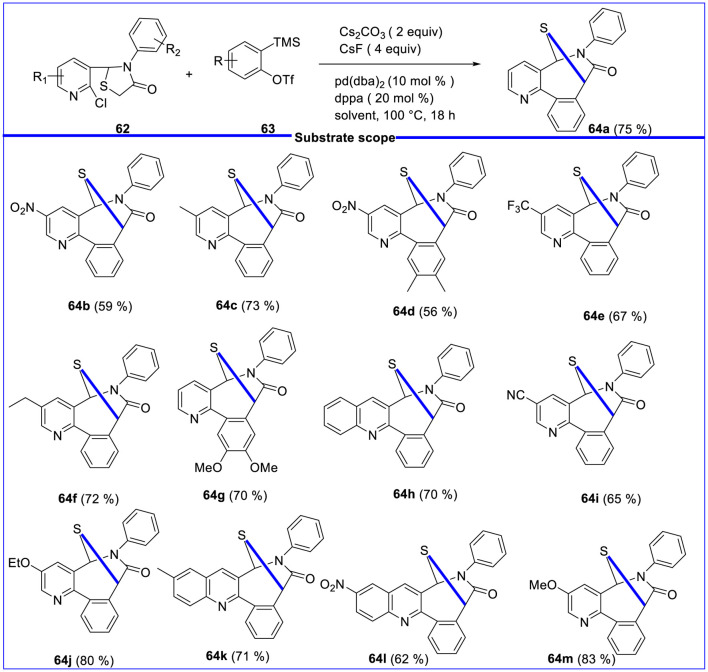
Synthesis of thio-bridged compounds via palladium (0)-catalyzed aryne annulation.

Morja *et al.* described the synthesis of thio-bridged compounds using aryne annulation catalyzed by palladium (0) (Scheme 22) (Morja and Chikhalia, 2023). The extent of this transformation was methodically investigated under ideal reaction circumstances by producing a small library of thio-bridged compounds using aniline, o-(trimethylsilyl) aryl triflates, and differently substituted 2-chloro 3-formyl pyridines. 2-Chloro 3-formyl pyridine with methyl or ethyl groups on the three-position produced a yield of 72%–73%. Because of the electronic effect, substrates with electron-rich groups on the pyridinyl ring produced a higher yield than those with electron-poor groups. For this change, two potential routes—path **A** and path **B**—were examined.

**SCHEME 23:**
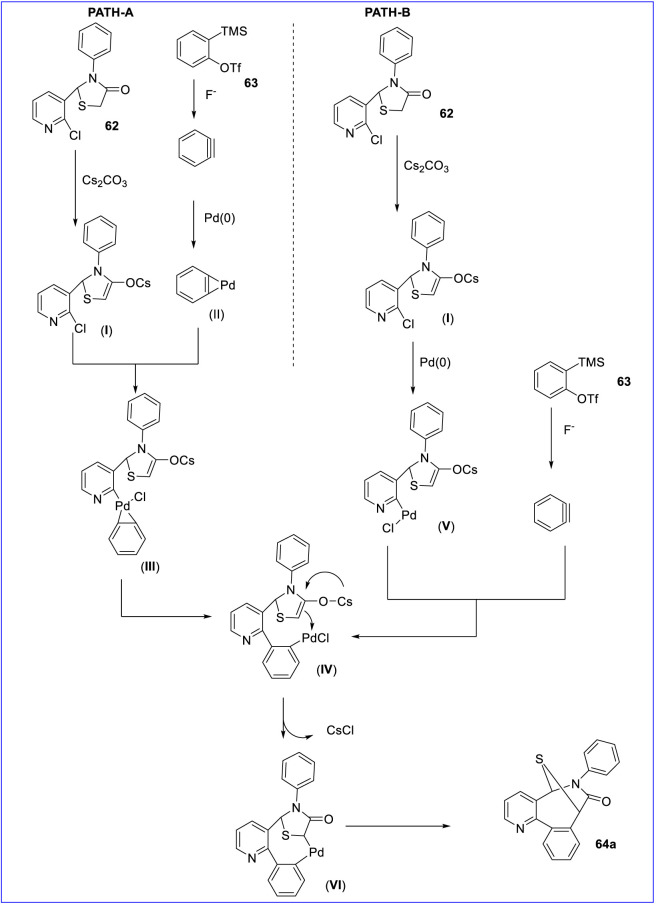
A plausible mechanism of thio-bridged compounds.

First, thiazolidine-4-one’s active methylene group is deprotonated with Cs_2_CO_3_ to produce enolate **I**. In path **A**, palladacycle **II** is created by oxidative cyclization of the aryne from the silyl triflate with pd (0) in the presence of a fluoride source. Aryl palladium **(IV)** complex **III** may result from the oxidative addition of enolate to palladacycle. After reductive elimination, Complex **III** may provide a new aryl palladium **(II)** intermediate **IV.** In contrast, process B entails the direct oxidative addition of Pd (0) to aryl halide to produce aryl palladium **(II)** intermediate **V**, which subsequently undergoes aryne carbopalladation to produce intermediate **IV**. Regardless of how intermediate **IV** is created, eight-membered palladacycle **VI** is created by subtracting CsCl from this intermediate. **VI** may undergo reductive elimination to produce the expected thio-bridged product and replenish the palladium (0) catalyst, completing the catalytic cycle (Scheme 23).

**SCHEME 24:**
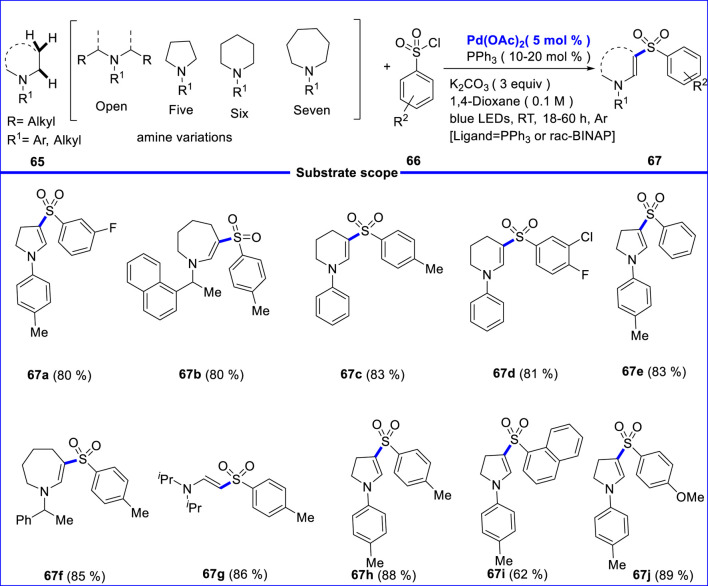
Pd-catalyzed dehydrogenative *β*-sulfonylation reactions using amines and aryl sulfonyl chlorides.

Muralirajan *et al.* reported the dehydrogenative sulfonylation of amines (**65**) (Muralirajan et al., 2023). The photocatalytic protocol was used on a variety of amines (**65**) and aryl sulfonyl chlorides (**66**) after optimization of the reaction conditions. Both electron-donating (ED) and electron-withdrawing (EW) substituted sulfonyl chlorides reacted efficiently with 1-(*p*-tolyl) pyrrolidine and 1-phenylpiperidine to give sulfonylated products in high yield and selectivity. Electronic effects had potent impacts on the reactivity of *N*-phenyl pyrrolidines ED substituents increased the yield of products, whereas EW group decreased the efficiency because the energy of abstraction of *α*-hydrogen has increased. *N*-phenyl and open-chain (*N*, *N*-diethyl) phenyl amines (seven members each) provided yields of vinyl sulfonylated products in 81% and 62% yield, respectively, indicating that electron-rich amines are preferentially oxidized to the dehydrogenated product. Triethyl-, butyl-, and hexylamines (aliphatic amines) were also capable of producing stable vinyl sulfones in high yield. The procedure worked well with unsymmetrical tertiary amines (e.g., diisopropylethylamine, *N*-methyl-piperidine and substituted azepanes) without affecting adjacent regio-centers, depending on the ring size (Scheme 24).

**SCHEME 25:**
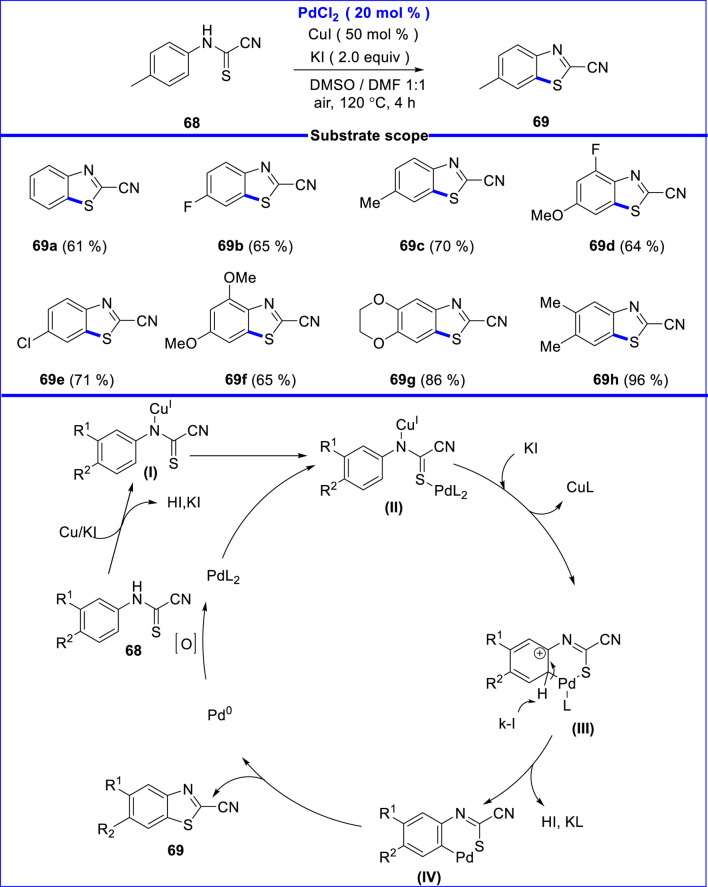
Pd-catalyzed/Cu-assisted intramolecular C-S bond formation.

Broundic *et al.* revealed the synthesis of 2-cyanobenzothiazoles (**69**) utilizing 2.0 equiv. of an inorganic additive (KI) and Pd-catalyzed and Cu-assisted intramolecular C-S bond formation from *N*-Arylcyanothioformamides (**68**) in the presence of air (Broudic et al., 2022)The base-assisted synthesis of the Cu(I) thioamidate **(I)**, which favors the coordination of Pd(II) with the sulfur atom to create the intermediate **(II),** is most likely what starts the reaction. The palladacycle **(IV),** which undergoes reductive elimination to produce the desired 2-cyanobenzothiazole (**69**) and Pd (0), can be re-oxidized by atmospheric oxygen after a de-protonative metalation step that forms a sterically hindered transition state driving the regioselectivity **(III)** (Scheme 25).

### 1.5 Miscellaneous catalysts

These transition metals provide selective access to thioethers, thioesters, disulfides, and xanthates over a wide range of substrates with good yield, emphasizing their significance in sustainable synthesis, medicinal chemistry, and sophisticated sulfur-containing molecules. Molybdenum catalysts effectively facilitate the dehydrative and borrowing-hydrogen thio-etherification of primary and secondary alcohols, benzylic alcohols with thiols, whereas iridium catalysts under visible-light conditions enable the conversion of sodium aryl sulfinates into valuable thioesters. Rhodium catalysis facilitates carbene-mediated rearrangements of allylic and propargylic sulfides, whereas chromium provides a cost-effective, ligand-free route to unsymmetrical disulfides via reductive cross-coupling of unactivated alkyl electrophiles. Manganese-mediated radical click strategies allow rapid access to xanthates, zinc catalysts promote bioinspired C–S *σ*-bond metathesis of alcohols to thioethers, and scandium catalysis supports tandem conjugate addition pathways to functionalized benzylic thioethers.

### 1.6 Molybdenum catalyst

**SCHEME 26:**
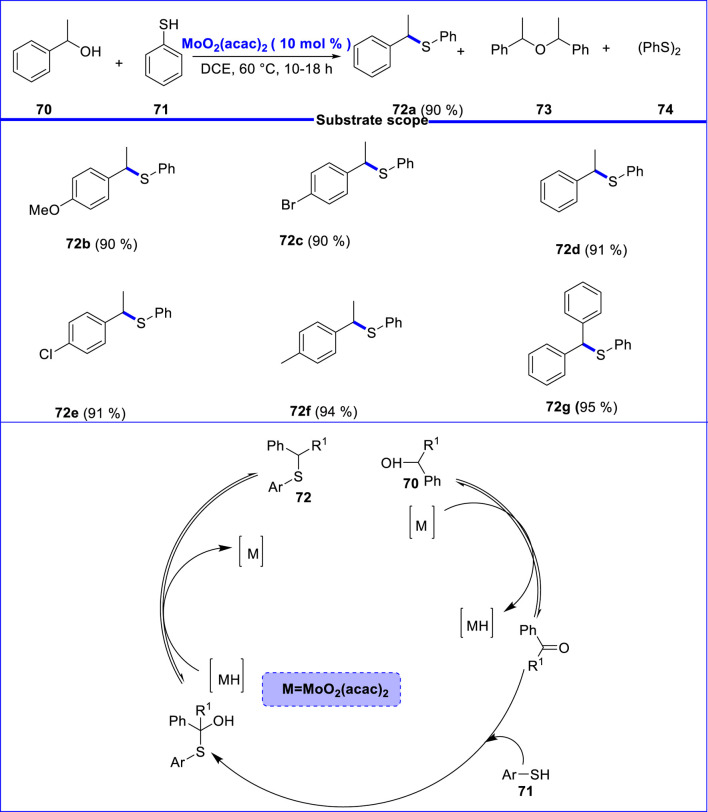
Thio-etherification of alcohols and thiols.

Alcohol’s dehydration transition into useful end products has drawn a lot of interest in chemical synthesis (Haibach et al., 2013; Swamy et al., 2009) Mo (VI) catalyzed dehydrative reactions converts alcohols into symmetrical/unsymmetrical ethers and thioethers under mild, water generating conditions with secondary benzylic alcohols forming symmetrical ethers that reacts with primary alcohols to give unsymmetrical products. There were trace amounts of symmetrical ether and disulfide by-products in addition to the intended thioether. They are probably the result of conflicting thiol oxidation and alcohol self-coupling reactions. However, the catalytic system demonstrated good selectivity for the production of the desired C–S bond. Rajmani Singh *et al.* revealed that the dehydrative synthesis of C-S bonds was selectively facilitated by molybdenum (VI) dioxo (acetylacetonate)_2_ through the thio-etherification of thiols and benzylic secondary alcohols (Singh et al., 2020). Alcohol (**70**) is oxidized to matching ketone **C** by reducing molybdenum, which is subsequently attacked by thiols to produce intermediate **D**, which releases the water molecule to supply corresponding thioether products (**72**). This is the reasoning for the thioether production mechanism. Additionally, this technique demonstrated how alcohol’s hydroxyl functional group may be activated to produce desired compounds in large quantities (Scheme 26).

**SCHEME 27:**
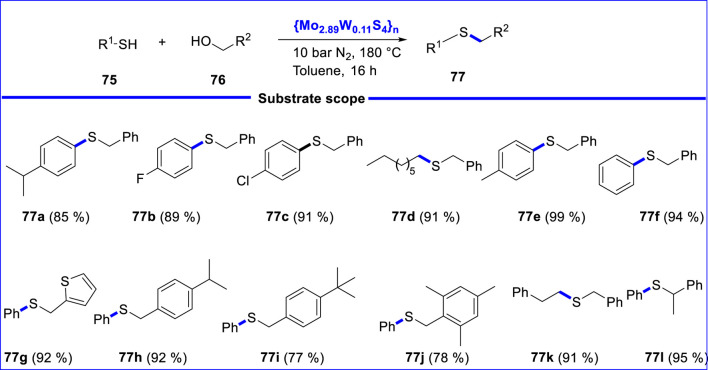
Thioetherification of alcohols through molybdenum sulfide.

Addition of the heterogeneous catalysts in the process of thio-etherifying alcohols has emerged as a viable and sustainable approach to the production of thioethers. Rodenes *et al.* reported the Thio-etherification of Alcohols (**76**) through molybdenum sulfide (Rodenes et al., 2025). Here, a unique synthesis process of isostructural molybdenum and tungsten sulfide molecular complexes of M (M = Mo) cluster coreswere used to create an original catalyst by alloy engineering the catalysts’ basal planes. From the perspective of green and sustainable chemistry, borrowed hydrogen thio-etherification of alcohols has emerged as a highly significant and appealing method in recent years (Corma et al., 2018; Hima et al., 2023; Shimizu, 2015). In order to avoid using precious metals, cobalt-molybdenum sulfide unsupported materials were been reported (Sorribes et al., 2017; Sorribes et al., 2018), they proved to be excellent catalysts for the formation of C-S bonds in thioethers via the borrowing hydrogen strategy by reacting thiols (including hydrogen sulfide) with primary and secondary alcohols (Sorribes and Corma, 2019) (Scheme 27).

### 1.7 Iridium catalyst

**SCHEME 28:**
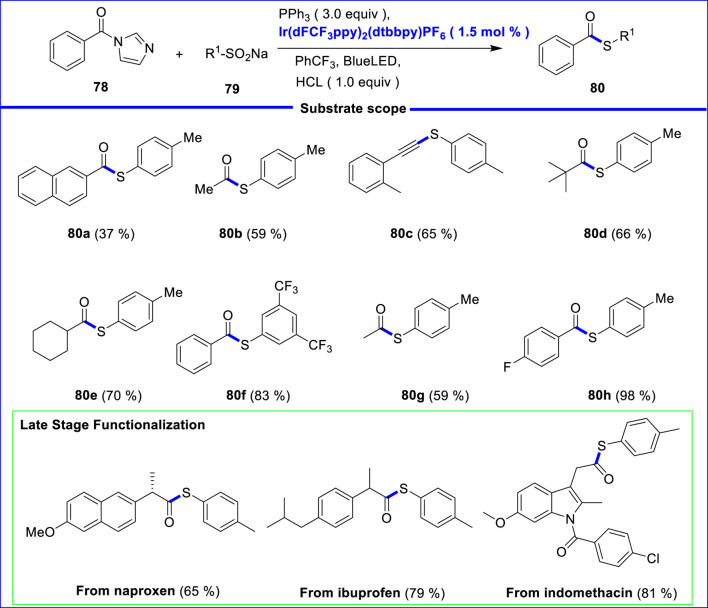
C–S bond construction via reductive coupling of phthalimide derivatives.

Thioesters (Tokuyama et al., 1998) and thio-alkynes (Gray and Wilden, 2016) function as important synthetic intermediates. Chen *et al.* transformed the sodium aryl sulfinates (**79**) into thioesters and thio-alkynes using a visible-light-driven reaction using phosphine as a mediator (Chen et al., 2025). Sodium sulfinates, which are stable and easy to handle, have become effective sources of sulfur. A phosphine-mediated deoxy-functionalization of the sodium aryl sulfinates driven by visible light is introduced to get thioesters and thio-alkynes formed with acyl imidazoles (**78**) and iodo-alkynes. The process is tolerant to different substituents and functional groups, and only para-chloro, trifluoromethyl, and methoxy analogs were reduced in yield by minor amounts. The naphthyl and heteroaryl sulfinates, including thiophenes, are also effective in the optimized conditions (Scheme 28).

### 1.8 Rhodium catalyst

A class of planar six-membered ring compounds with conjugated carbonyl, alkene, and diazo groups is known as di-azo-quinones (Sander et al., 1993). The quinoid carbene’s special structure gives it particular reactivity characteristics, such as disposition for aromatization (Kitamura et al., 2011; Kitamura et al., 2014; Sundberg et al., 1988; Zhang et al., 2015b), strong electrophilicity, and quinone-like hydrogen atom transfer (HAT). Yan *et al* described the chemo-selective rearrangement of sulfur ylide reactions from allyl/propargyl sulfides (**82**) and di-azo-quinones (**81**) (Yan et al., 2020) The rearrangement of a range of allylic sulfides with di-azo-quinone was obtained in 41%–96%. Various allyl replacements (mono-, di-, and cyclic) were generally well-tolerated. The rearrangement also takes place with lactone-based allylic sulfides and cyclic ketone-based allylic sulfides, producing quaternary-centered products with high efficiency.

**SCHEME 29:**
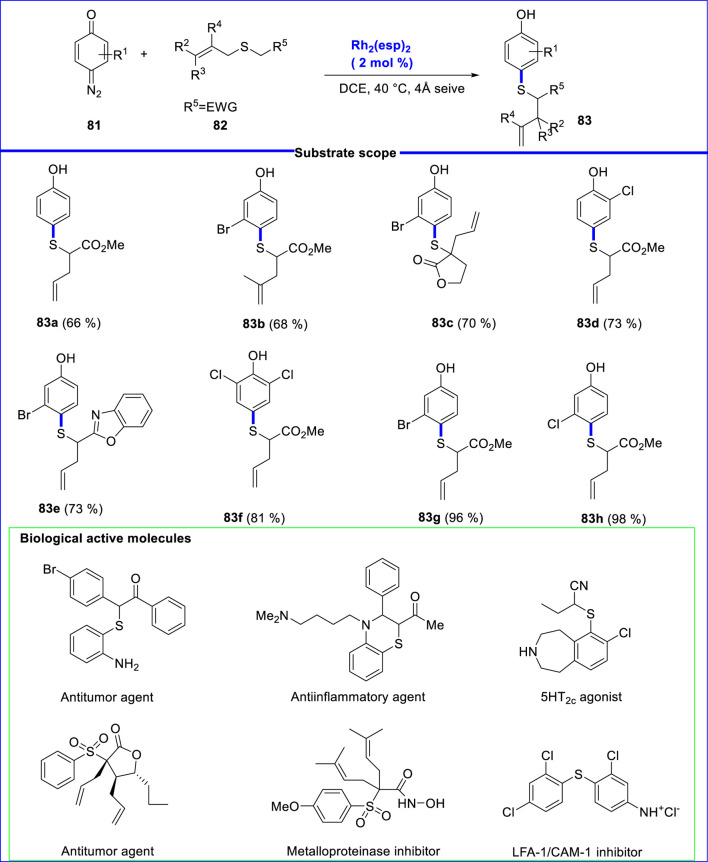
Chemoselective rearrangement reactions of sulfur ylide derived from diazoquinones and allyl/propargyl sulfides.

Other diazoquinones (meta-, ortho-, and disubstituted derivatives) are useful carbene precursors in the rearrangement catalyzed by Rh (II) with an overall yield of 60%–98%. The low solubility of diazoquinone in 1, 2- dichloroethane gives moderate yields with unsubstituted diazoquinone. The reaction with diazoquinone with an ester group is a reaction conducted at a lower rate, presumably because the electron-withdrawing group of the ester group decreases the susceptibility of the compound to ester-metal catalyzed decomposition (Doyle et al., 1998) (Scheme 29).

### 1.9 Chromium metal

**SCHEME 30:**
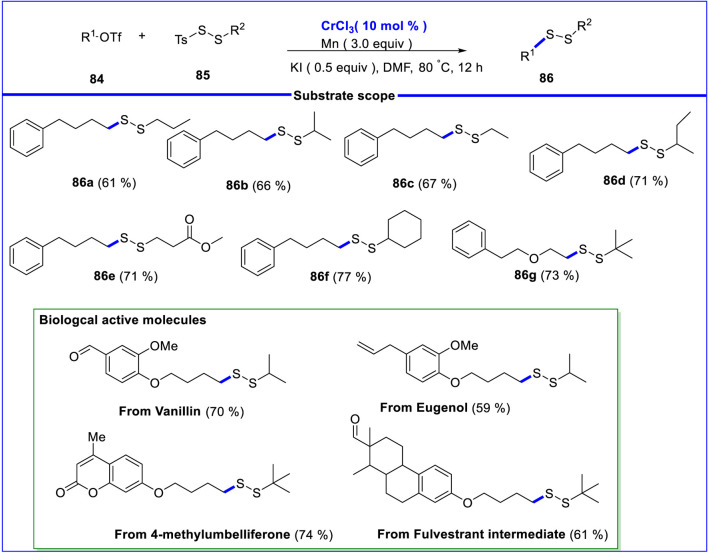
Synthesis of disulfide via chromium-catalyzed reductive cross-coupling.

Zhang *et al.* developed chromium-catalyzed reductive cross-coupling to synthesize C-S bonds from un-activated alkylating agent (**84**) (Zhang et al., 2024). An efficient form of C-S bond building, a ligand-free, low-valency chromium (III) catalyzed manganese reductive cross-coupling of alkyl sulfonates and chlorides with trisulfide dioxides. The technique has extensive substrate coverage, functional group tolerance, and is capable of yielding unsymmetrical disulfides in high yields under mild conditions. Due to its cheapness and the fact that it is not toxic, chromium is considered a sustainable substitution of other transition metals. No ligands are required in the reaction and the starting materials are readily available, which offers an accessible solution to useful disulfide products that can be used in medicinal, biochemical, and synthetic chemistry (Scheme 30).

### 1.10 Manganese metal

**SCHEME 31:**
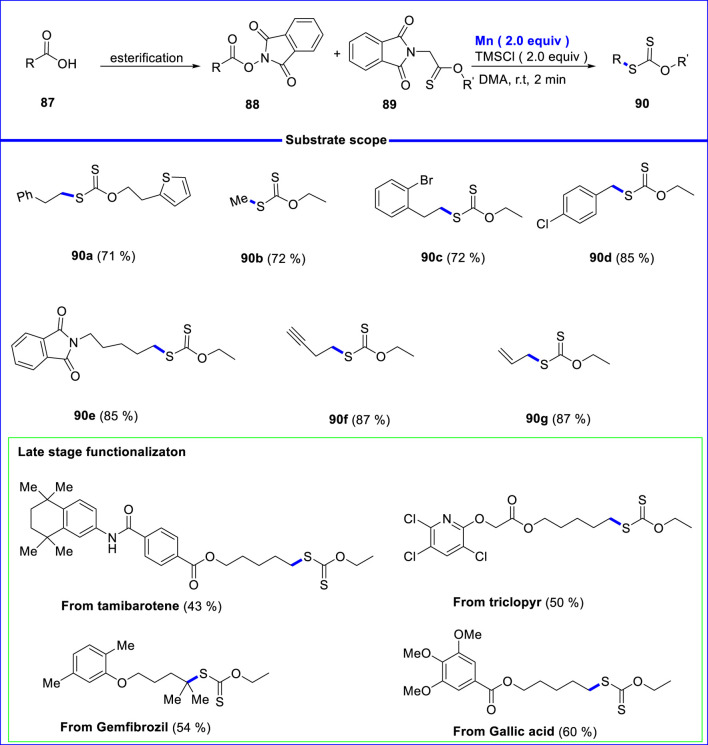
Synthesis of *O*-alkyl thiocarbonate via reductive coupling of phthalimide derivatives.

Radical chemistry has now become part of the organic synthesis, and there is still a push to find new ways of generating and using radicals (Qiu et al., 2024; Tang et al., 2020b). The selective and fast synthesis of small molecules under mild conditions by means of efficient connection of small molecules through heteroatoms is facilitated by click chemistry which allows the production of a wide variety of compounds (Tang and Becker, 2014). Qiao *et al.* described the reductive coupling of phthalimide compounds to create C–S bonds via the radical click reaction (**88** and **89**) (Qiao et al., 2025). Different complex molecules such as drug derivatives and natural products were subjected to efficient decarboxylative xanthylation with decent yields. The reaction was tolerant to different functional groups and was not highly electronic sensitive, but ortho substituents reduced the yield a bit due to steric effects (Scheme 31).

### 1.11 Zinc catalyst

**SCHEME 32:**
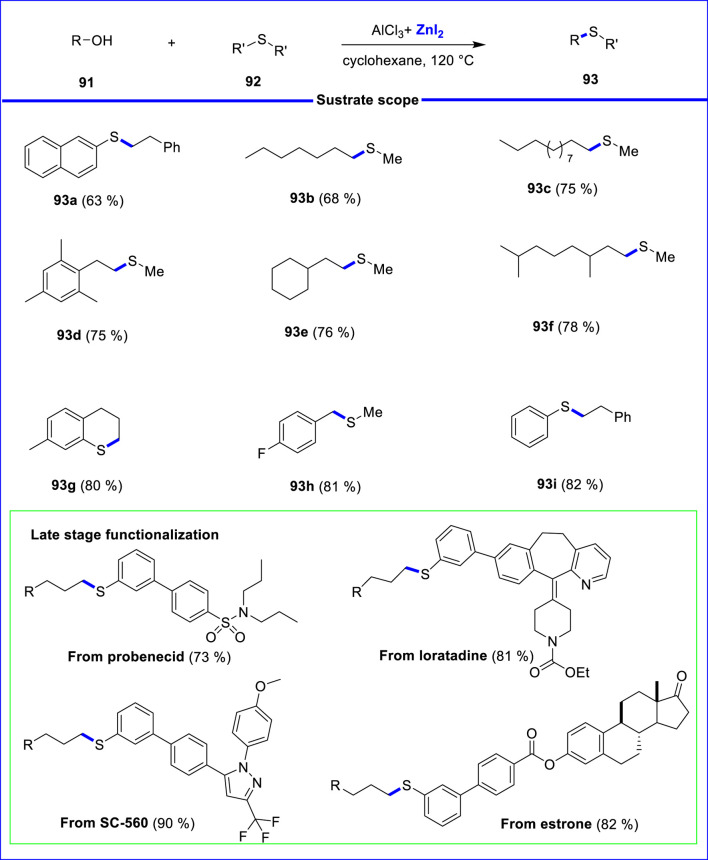
Biomimetic C S *σ*-bond metathesis reaction between alcohols and thioethers.

Hongmei *et al.* reported the sulfide synthesis via a bioinspired C-S σ-bond metathesis (Liu et al., 2025). Under optimized conditions, the C–S metathesis had a wide substrate range. The aliphatic alcohols of different chain lengths produced alkyl methyl sulfides in moderate to excellent yields whereas the cyclic secondary alcohols produced products in 70% yields. Alcohols in tertiary form were nonreactive. Et_2_S also was effective and unsymmetrical thioethers reacted selectively at the less sterically hinder side. Benzyl, substituted benzyl, 2-phenylthanol and 2-naphththanol derivatives were synthesized to moderate to good yield (up to 52%) using aromatic alcohols without any side reactions. Allylic alcohols that were electron-withdrawing group substituted produced products of metathesis with a yield of approximately 50% (Scheme 32).

### 1.12 Scandium metal

**SCHEME 33:**
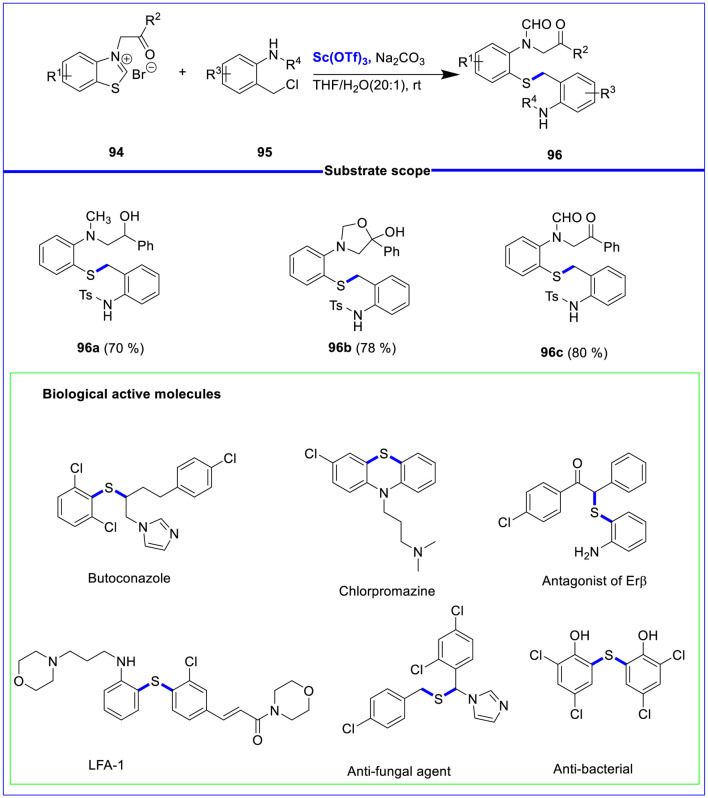
Synthesis of benzylic thioethers.

Chenyi *et al.* reported two amino benzylic thioethers (**96**) by a hydrolytic tandem 1,4 conjugate addition reaction of *N*-(2-chloromethyl)aryl amides (**95**) and benzothiazolium bromides (**94**) (Li et al., 2025)**.** The target products were produced in good yields with 5-Br and 5-Cl atoms on the aromatic ring. Under ideal reaction conditions, electron-donating groups, particularly methyl, or electron-withdrawing groups, namely, Cl, Br, or F atoms at the four-position of the benzene ring, produced the products in 65%–93% yields. Additionally, substrates having alkyl and halogen substituents at the benzene ring’s two or three positions could also be easily converted into the corresponding products in yields of 65%–89% (Scheme 33).

## 2 Conclusion

This review highlights the recent advancement in transition-metal-catalyzed C–S bond formation, underscoring its central importance in modern organic synthesis, medicinal chemistry, and late-stage functionalization of complex molecules. Classical metals as palladium, nickel, and copper, offer broad substrate scopes through ligand design and diverse sulfur sources, including thiols, disulfides, sulfinates, and elemental sulfur. Furthermore, miscellaneous and earth-abundant metals as molybdenum, iridium, rhodium, chromium, manganese, zinc, and scandium, have significantly expanded the synthetic landscape to thioethers, thioesters, disulfides, xanthates, and related sulfur-containing motifs. These transformations often operate under mild, ligand-free, photochemical, or radical conditions, emphasizing sustainability, cost-effectiveness, and functional-group tolerance. Notably, many of the discussed methodologies demonstrate excellent compatibility with drug-oriented molecules, highlighting their late-stage diversification. Despite remarkable advances in transition-metal catalysed C-S bond formation, several challenges remain, including the development of more sustainable catalytic systems, improved functional-group tolerance lower catalysts loading and broader applicability towards complex bioactive molecules. Future research should focus on earth-abundant metal catalysts, greener reaction conditions and mechanistic understanding to further enhance the efficiency and practicality of C-S bond forming methodologies.
